# Implementation of the cognitive apprenticeship model for enhancement of advanced searching skills in a pharmacy academia rotation

**DOI:** 10.5195/jmla.2022.1108

**Published:** 2022-01-01

**Authors:** Hilary Jasmin, Kenneth Hohmeier, Christina Spivey

**Affiliations:** 1 hjasmin@uthsc.edu, Assistant Professor, Health Sciences Library, University of Tennessee Health Science Center, Memphis, TN; 2 khomeie@uthsc.edu, Associate Professor, Department of Clinical Pharmacy and Translational Science, University of Tennessee Health Science Center, Memphis, TN; 3 cspivey@uthsc.edu, Associate Professor, Department of Clinical Pharmacy and Translational Science, University of Tennessee Health Science Center, Memphis, TN

**Keywords:** instruction, pharmacy, rotation, cognitive apprenticeship

## Abstract

**Background:**

Pharmacy students are primarily taught literature searching skills didactically during their Doctor of Pharmacy curriculum. To effect change in the area of advanced literature searching skills, a pharmacy librarian joined with two Advanced Pharmacy Practice Experience (APPE) preceptors to design and implement a crash course on applied systematic searching skills for a cohort of four students.

**Case Presentation:**

Through the cognitive apprenticeship model, a Systematic Searching Crash Course (SSCC) was implemented among a cohort of four academic APPE students. Students developed search strategies using controlled vocabulary and free text, translated their searches into multiple databases, and used citation management software to build libraries of evidence. Additionally, the cohort blindly peer reviewed each other's search strategies, wrote literature reviews, and finally conducted a search together without input from the pharmacy librarian.

**Conclusions:**

Review of the pre-/post-course self-assessment taken by the cohort indicates the SSCC is a success in terms of improving student confidence in accessing and synthesizing primary literature. As the crash course is further refined and implemented, there may be more opportunity to embed the course into didactic curriculum and residency programs and to potentially reproduce it for other health science disciplines.

## BACKGROUND

Involvement of a librarian in the process of improving research skills is a dynamic opportunity to further understand how students in health professions programs such as pharmacy are retaining librarian instruction from year to year of their curriculum. As liaison librarians advocate for ownership in curricula, there is a need to demonstrate what librarian instruction can contribute to student learning. It is often the onus of librarians to insert themselves into these spaces and demonstrate their value in a way that lightens the load of course directors [1].

Most available evidence on preparing pharmacy students as researchers lives in didactic- and laboratory-based studies, but there is a need for application of these skills for the sake of true competence on the part of the learner [2]. Evidence supports longitudinal training on literature searching skills through tying didactic and experiential components together. However, previous studies note that student learning of librarian instruction is not well retained, possibly due to its sporadic nature [3].

In the environment of Doctor of Pharmacy (PharmD) education, an Advanced Pharmacy Practice Experience (APPE) (i.e., clinical rotations, clerkships) is a required learning experience in a practice-based or real-world setting precepted by experts in that setting, which builds on didactically delivered content and typically runs for one month before students rotate to the next APPE [4]. Learners are typically in their third or fourth (i.e., final) year of the PharmD program. Regardless of whether the learner has an interest in pursuing a career in research or academia after their PharmD, it is an expectation of the profession that upon graduation a pharmacist must have the skillset to work interprofessionally in retrieving and synthesizing primary literature in order to affect patient care [4,5].

APPE learning involves unique challenges, including the time-limited nature of a rotation. The ability to be oriented, conduct a study, and write up results in four weeks is highly challenging and nearly impossible if the rotation student enters an academic- or research-focused APPE with no research experience [6]. This contradicts the priority the profession places upon scholarship. As part of the Core Entrustable Professional Activities (EPA) for New Pharmacy Graduates, it is expected that the PharmD is an “Information Master,” meaning they are able to employ evidence-based research to answer drug information questions and enhance patient care in a thoughtful, critical manner [5]

Considering the COVID-19 pandemic and rapid move to remote teaching, the University of Tennessee Health Science Center (UTHSC) College of Pharmacy (COP) created a task force of pharmacy faculty to study and disseminate research related to the subsequent impact of the shift to online education on the curriculum, students, and faculty. To facilitate this effort, two members of the task force worked with the Office of Experiential Learning within the COP to provide an academic APPE in May 2020 in which students would conduct a series of systematic literature searches related to the shift to remote teaching in collaboration with the college's library liaison.

Given the challenges of remote teaching, the pharmacy librarian found the cognitive apprenticeship (CA) model facilitated learning for the APPE students. This model is a pedagogical approach that prioritizes the visual application of skills performed by the expert for the benefit of the learner. Its foundations include concepts borrowed from other theory and pedagogy, including the zone of proximal development (ZPD), situatedness, and scaffolding [7,8]. In sum, these theories together form the notion that a learner should be introduced to new skills in an order that takes into account the learner's current skillset, within an environment that allows them to directly apply new knowledge within their given trade or profession, and with more autonomy given to the learner over time. This constructivist approach comes naturally to the profession; librarians often demonstrate databases, walk through tools, and construct search strategies live in front of a classroom [7]. In one-on-one opportunities to work with students on application of skills rather than didactic lectures, librarians can have more effect on establishing best practices and integrating information literacy skills [8,9]. Such an experience may prove to be an “opening” for librarians to bridge skill gaps between lecture-based instruction in the classroom and practice-based application.

With these barriers and opportunities in mind, two rotation preceptors collaborated with their liaison librarian to enhance an academic APPE by scaffolding in advanced searching skills through cognitive apprenticeship to strengthen four student pharmacists' confidence as members of the scholarly community.

## CASE PRESENTATION

The program description outlined below followed the guidelines for reporting evidence-based practice educational interventions and teaching (GREET) [9]. GREET breaks down the intervention by the following: why, what, who, how, where, when and how much, adjustments, and how well.

The librarian liaising to the college of pharmacy developed the Systematic Searching Crash Course (SSCC) and acted as a coinstructor with two preceptors to invest time and priority into developing advanced literature searching skills in an academia rotation. The supporting theory, the cognitive apprenticeship model, was employed for direct application of skills from the expert (the librarian) to the apprentice (the student pharmacists) [10]. The learning objectives for the SSCC were as follows.

Librarian-curated:

- Construct a term-harvesting spreadsheet where searches are developed and stored
- Build a tested, completed search strategy
- Translate the final strategy into multiple databases
- Create a library of relevant scholarship to build a literature review

Mapped to college competency standards:

- Identify, refine, and categorize the question(s) to be researched
- Develop a search strategy to retrieve pertinent information from appropriate sources
- Conduct a search using scholarly databases

Due to its fully online format, all material was virtually disseminated via Zoom. In addition to the syllabus and schedule, a shared folder with a Microsoft OneDrive spreadsheet was used to document all search strategies and term-harvesting practices. Additionally, Microsoft Teams and OneNote were employed for collaboration and discussion space. Videos on systematic searching from Yale's Harvey Cushing/John Hay Whitney Medical Library were suggested in the schedule of activities of the SSCC [11].

The first of three sessions of the baseline (i.e., traditional) model is a discussion of the student's research topic and a building out of terms in a spreadsheet. Controlled vocabulary is discovered through PubMed by way of MeSH terms, and free text is explained as the natural language found in scholarly articles. Additionally, systematic searching in comparison to the nonsystematic strategies learners used previously is discussed. Between the first and second session, the student completes the harvesting of terms in their spreadsheet, after demonstrations from the librarian in the first session. In the second session, the student's strategy for their search is tested in PubMed/MEDLINE. This is an opportunity for the student to see the myriad ways a term they think is appropriate may be pulling in irrelevant material and helps illustrate the trial-and-error process that comes naturally with searching. Once the strategy is ready, translation of the search for multiple databases is discussed. Primarily, additional databases used have included Elsevier's Scopus, Clarivate's Web of Science, the Cumulative Index for Nursing and Allied Health Literature (CINAHL), and the Cochrane Library. The librarian explains that translations are simply a reworking of the terms so the strategy is friendly to the new database while remaining as identical as possible to the completed PubMed search. In the third and final session, all translations are run and exported into EndNote, and deduplication, finding full text, and employing Cite While You Write are discussed. This last session is run by the student; the librarian gives them the power to share their screen, and the student runs all the searches and exports into their EndNote library. Upon completion, there is reflection on their comfort in databases compared to when the student began, and the librarian prompts the student to submit an annotated bibliography of deduplicated results to their preceptor as a deliverable demonstrating completion of the SSCC.

For the May 2020 academic APPE, a significant amount of modifications were made to enhance the experience, including multiple research topics. Given the COVID-19 experience, the students were assigned individual research topics revolving around remote learning as a topic of interest: instructor perceptions, student perceptions, tools and methods, and emergency transitions to remote learning. In addition to the traditional three sessions described previously, the cohort met individually with the librarian to discuss their searches, since each was working on a different topic. There were also follow-up meetings to discuss the use of EndNote while writing their literature reviews (Appendix 1). In the first group session, the librarian and student cohort developed a base search strategy on remote teaching and learning that would be incorporated into their individual searches (Appendix 2). Note that while this was a systematic searching course, the rigor of systematic review methodology was not the intent. Rather, the motive was to develop students' critical thinking around searching in a more exhaustive and intentional way.

The traditional model requires a deliverable of an EndNote library and an annotated bibliography after references have been deduplicated. Additionally, the May 2020 cohort conducted a blinded peer review of each other's search strategies, and each student wrote a five-to-seven-page literature review synthesizing their evidence. Finally, the cohort worked as a team to develop a search strategy using the skills they gained while working with the librarian. The librarian had no input on the search and assessed its quality after course completion.

Through their concerted efforts, the students were encouraged to expand their literature reviews into submitted articles for publication. This opportunity to obtain authorship and begin adding scholarship to their curricula vitae was an incentive for their efforts. At least one of the learners has made effort to publish their study.

The cohort felt overwhelmed by the accumulating due dates. In particular, the literature review was due one week after the annotated bibliography was compiled. As students had not written a literature review since their undergraduate studies, this was daunting for them, so the due date was delayed by one week.

None of the four students missed a session, but this was partially due to the librarian's ability to accommodate their schedules and change meetings as needed. Recordings of each Zoom meeting were posted to the Teams folder for students who wished to rewatch.

There were multiple points for assessment from both peers and instructors. After initial search strategies were developed, the librarian collected them into a spreadsheet, labeling them A, B, C, and D. Each student then used a OneDrive form to review the searches of the other three cohort members (Appendix 3). Students were additionally asked for any short answer feedback and if they believed the strategy was complete or needed further elaboration. This blind review provided an opportunity for each student to see how others were searching and receive feedback on their own search.

The final search conducted by the cohort at the end of the APPE was undertaken as a group effort without instruction or support from the librarian. This was done to assess retention of skills they learned at the beginning of the month. This final topic was a search for novel rotation/APPEs for remote care in emergency situations. This search was also compiled into an EndNote library, but the cohort did not write a literature review as the APPE was concluding.

Finally, students were asked to complete the first half of their pre-/post-course self-assessment before the first meeting. This assessment asks the student to rate themselves on a scale of absolute beginner, novice, developing, or competent in searching systematically; using controlled vocabulary; creating a reproducible, multiconcept search strategy; translating a search for reproduction in multiple databases; and building a library of references in a citation manager (Appendix 4). They completed the self-assessment a second time at the end of the month.

## DISCUSSION

The aim of the SSCC for student pharmacists on an academic APPE rotation as part of their PharmD training program was to leverage interprofessional collaboration between library and pharmacy faculty to build upon literature searching skills. Student autonomy increased with each subsequent week of the month-long APPE, in line with their growing skills. It is important to note the outcome sought was not for student pharmacists to be able to conduct a completed systematic review; rather, a rigorous systematic search was the planned product. This approach is aligned with the theory of CA. Based in constructivism, CA postulates that training students on certain skills is best accomplished through guided experiences overseen by an expert who first models and then coaches students through a given process [12]. Its core constructs include 1) situatedness, 2) peripheral participation, 3) guided participation, and 4) community of practice. Pharmacy EPAs of the APPE are mapped to these constructs (Table 1).

**Table 1:** Entrustable professional activities (EPA), zone of proximal development (ZPD), and scaffolding mapping

Levels	Pharmacy EPA	Scaffolding	Potential future abilities.
	Information Master Domain: Use evidence-based information to advance patient care.		
5—Supervision provided by the trainee to more junior colleagues	Work collaboratively with other students to practice and teach skills related to retrieving and analyzing scientific literature	Conduct final search collaboratively with other APPE students to teach each other and share gaps in understanding	
4—Supervision at a distance and/or post hoc	Analyze retrieved literature and synthesize to answer patient, research, or drug information question	Individual EndNote library creation, annotated bibliography creation, and literature review on assigned topic	
3— Execution with reactive supervision, i.e., on request and quickly available	Retrieve scientific literature without direct guidance from an expert	Guided participation in performing individual searches, with as-needed assistance from librarian and weekly debrief with pharmacy faculty	
2— Execution with direct, proactive supervision	Retrieve scientific literature with guidance from an expert	Guided participation on use of search platforms and EndNote	
1— Observation but no execution, even with direct supervision	Identify the process of retrieving scientific literature	Direct instruction and peripheral participation on systematic literature searching basics; live session watching search performed on general search term	Current abilities.

In designing the rotation, participating faculty noted the general lack of literature searching skill application by UTHSC COP student pharmacists. General APPE literature search skill development occurs via pharmacist-student interaction and usually within a patient care setting. Although this reflects a more true-to-practice learning environment, it may not adequately bridge content taught in the classroom with the more practical application found in the patient care environment. According to CA, this gap between current student skills and ideal pharmacist literature search expertise is known as the ZPD, which is a middle area between where the student's current abilities lie and potential future expertise [8]. The use of CA to foster development in this zone has been explored at length [13–15].

*Situatedness* is active learning with the genuine task or within the genuine setting [7]. In this experiential learning rotation, students were situated to a virtual environment where they were performing a task with full access to university library resources and using virtual technology used by faculty and staff when collaborating across distant campuses. In this way, the learner was immersed in practice. Learners had to manage their time, troubleshoot technology barriers, and deal with natural environments that are difficult to simulate and teach in a classroom. For instance, in addition to being tasked with literature search milestones, students were also required to complete ancillary but related assignments such as interviewing a faculty member of their choice and submitting a written reflection. Management of multiple tasks without a traditional classroom or lab-based structure can be challenging for students [16,17]. Students also articulated this issue in their daily standing meetings and weekly debrief with preceptors:

It's been beneficial because me and a lot of other students know we need to do this kind of searching but it's easy to get really frustrated when you try it in the classroom. I had heard about MeSH terms before but I would say I could use it but I wasn't comfortable using it. I'm now comfortable with it. (Student A, Weekly Debrief 1)

*Peripheral participation* is a learner being engaged in observation of a genuine task from the periphery. Early in learning within the ZPD, one does not need to be actively engaged in a task to be a legitimate learner until they have witnessed and understood the big picture as demonstrated by an expert (i.e., modeling). In this case, this was facilitated by the librarian who performed an initial search using live, virtual technology (e.g., screen sharing) after direct instruction on what she was going to do to perform the search. Students noted it was helpful to see this modeled first and that it connected ideas previously learned during a drug information course but which were not fully assimilated by the student.

… [watching the librarian search] it really shocked me how inefficient and ineffective my searches were. What you find is only as good as the question you ask. How the databases work there's a correct way versus just a keyword search—you'll find something for sure, but you may not find the most real net or as much information as you could have. (Student B, Weekly Debrief 1)

Students specifically commented on how peripheral participation exposed gaps in learning via the traditional, practice-based literature search environment of a patient care setting. This aligns with the preceptor a priori hypothesis that this type of learning was not properly aligned with the students' ZPD.

When she went over how to do a systematic search I had realized even though I was doing the advanced search and using MeSH search on my own last month I really wasn't doing it correctly and so wasn't pulling all of the studies I should have … It probably impacted the quality of my presentation. (Student C, Weekly Debrief 1)

*Guided participation* follows from peripheral participation and involves students performing smaller parts of the larger process of what was modeled individually with coaching by preceptors. A scaffolding approach was used whereby preceptor support was withdrawn slowly and in accordance with learner growth across the stages of skill set development. Specifically, during both librarian-facilitated sessions and weekly pharmacy faculty-facilitated sessions, faculty monitored cognitive load for each student. If cognitive load was exceeded, further scaffolding in the form of separate one-on-one facilitation (email, telephone, virtual meetings) was provided. Some students progressed through the entire APPE without additional support, while others needed more frequent touchpoints.

Students within the guided learning stage of the APPE noted the recursive nature of learning while within their ZPD. They were provided incrementally more difficult tasks related to the literature review until finally they reached a point of internalizing search strategy for a particular search, only to start anew within the ZPD when introduced to a new search request. For example, one student who was performing an initial search assignment reflected on their need to rewatch a recorded meeting with the librarian where peripheral participation took place (librarian performing systematic search). The student rewatched the lecture and reached out to the librarian for further mentorship on the search skill. By scaffolding the content and recording its delivery, the student was able to self-assess gaps in their own skills as they progressed through their ZPD and move backward to the most proximal skill gained.

[I] re-watched the meeting with [the librarian] yesterday [and] watched Part 1 & 2 of the systematic searches videos, but am still feeling confused about how to perform a proper search … (Student C, Rotation Reflection Day 2)

Today, I had my individual meeting with her and we went over my translations and how to specifically translate for ERIC and CINAHL. We prepped everything for Friday's final search. We also reviewed the feedback from my peer reviews and agreed that my current search is fine the way it is. (Student C, Rotation Reflection Day 4)

Lastly, the concept of *community of practice* refers to the formal or informal grouping of individuals by common practice identity. The community of practice specific to these APPE students was of the profession of pharmacy. To facilitate professional development, students were asked to reflect on their week's progress and experiences related to the rotation. During a weekly debrief, pharmacy preceptors shared their own experiences with using literature search skills, integration into research and practice, and the value of these skills from a health professions perspective. These shared expectations, identity, and vocabulary created a deeper learning experience for the students and modeled a real-world practice environment. In practice, a pharmacist would use the collective expertise of their community to refine ideas and application of skills. Much of these weekly debrief sessions were sharing context of application of systematic searching skills to pharmacy practice and academia. Topics included responding to drug literature questions by prescribers, outlining and writing scholarly manuscripts, working with librarians, and deciding when to perform systematic literature searches. These sessions were also used to guide students through the process of synthesizing conclusions based on their literature searches and application to teaching student pharmacists.

Despite the limitation of a small cohort size, application of advanced literature searching skills through cognitive apprenticeship was a well-received method for rotation students and may advance current literature searching instruction practice. Working within a cohort provided an opportunity for team-based learning, and the virtual format provided a level of communication that may have been less accessible when all meetings would traditionally be face-to-face. This method of instruction in literature searching skills will continue to be explored in other levels of pharmacy education, as well as disciplines beyond pharmacy.

## DATA AVAILABILITY STATEMENT

Data associated with this article are available in the University of Tennessee Health Science Center Institutional Repository at https://dc.uthsc.edu/hslfacpubs/1/.

## SUPPLEMENTAL FILES

**Appendix 1** Schedule of activity mapped to scaffolding of learner support

**Appendix 2.** Student search strategies

**Appendix 3.** Questions from blinded peer review of search strategies

**Appendix 4.** Self-assessment results
