# Supplementary material for: Implementation of the cognitive apprenticeship model for enhancement of advanced searching skills in a pharmacy academia rotation
Source: J Med Libr Assoc. 2022 Jan 1;110(1):119–25. doi: 10.5195/jmla.2022.1108 (PMC8830337; doi:10.5195/jmla.2022.1108)
Supplement: Supplementary file 1 — Appendix 1 Schedule of activity mapped to scaffolding of learner support [file jmla-110-1-119-s01.docx]

**Appendix 1. Schedule of activity mapped to scaffolding of learner support.**

Before we meet:  Complete pre-survey on searching confidence

| Session | Agenda | Tasks to complete before next session | Scaffolding Structure |
| --- | --- | --- | --- |
| May 4 | — Discuss systematic searching and what that means  — Discuss document sharing and expectations  — Begin exploring the research topic and developing our concepts  — Go over peer review assignment | — After identifying all relevant concepts, watch this video from Yale’s medical library concerning MeSH terms.  — With those topics, identify Mesh terms in PubMed and add them to the spreadsheet. Next, watch this video from Yale’s medical library concerning keyword searching.  — After both videos, all our concepts should have Mesh terms and keywords developed in the term harvest spreadsheet. **Email the librarian your finished search strategy by Tuesday, May 5, at 12PM.** | Direct instruction and peripheral observation |
| May 5 | *no meeting*  — Blinded search strategies will be disseminated. Using this form, peer review the search strategies that are not yours. Ex.: if your search strategy is coded as “C,” use the form three times for “A,” “B,” and “D.” |  | Initial guided participation (Preceptor>>learner) |
| May 6 | — Test our “general” search strategy, determine weaknesses or gaps  — Discuss search translation  — Discuss peer review feedback | — Install EndNote  — Watch this video from Yale’s medical library concerning search translation.  —Work on translating searches for Scopus, ERIC, and Web of Science (WoS).  — I will send separately the peer feedback on your search strategy. Respond to the feedback in the document provided. |  |
| May 7 | *Individual meetings to discuss search strategies before translations are fully realized* |  | Early-mid guided participation  (Preceptor>learner) |
| May 8 | — Run our searches through PubMed, Scopus, ERIC, and WoS  — Export our results into EndNote  — **Share EndNote library with the librarian via email**  — Discuss remainder of rotation deliverables | — Deduplicate references in EndNote. When finished, export annotated bibliography to a Word doc and upload to Teams. |  |
| May 11 | — One-on-one meetings to discuss individual searches, EndNote libraries, Cite While You Write, and next steps for literature review due on May 15.  — **Annotated bibliography due in Teams** | — With EndNote library of citations, conduct literature review on assigned topic while employing Cite While You Write. | Mid-late guided participation (Preceptor<learner) |
| May 15 | — **Literature review due in Teams** (pushed back one week)  — Assign “final” search to be done individually without librarian input | — Conduct final search on your own, using the same methods we employed at the beginning of the month. Create a new spreadsheet to document your searches and translations. You will need to have a spreadsheet and an EndNote library of results as deliverables. |  |
| May 22 | — **Final search (spreadsheet and EndNote library) due in Teams** (pushed back one week) | — Complete post-survey on searching confidence | Late guided participation  (Preceptor<<learner) |
