# Supplementary material for: Implementation of the cognitive apprenticeship model for enhancement of advanced searching skills in a pharmacy academia rotation
Source: J Med Libr Assoc. 2022 Jan 1;110(1):119–25. doi: 10.5195/jmla.2022.1108 (PMC8830337; doi:10.5195/jmla.2022.1108)
Supplement: Supplementary file 2 — Appendix 2. Student search strategies [file jmla-110-1-119-s02.docx]

**Appendix 2. Student Search Strategies**.

Base strategy (attached with AND to individual topics):

("Education, Distance"[Mesh]) OR ("distance education" OR "remote learning" OR "remote teaching" OR "distance learning" OR "distance teaching" OR "remote education" OR "satellite campus")

Individual strategy: Instructor perception

("perception" OR "impression" OR "knowledge" OR "viewpoint" OR "approach" OR "attitude" OR "feeling" OR "opinion" OR "conceptualization" OR "thought" OR "judgement") OR ("Attitude"[Mesh]) OR ("Perception[Mesh]") AND ("faculty" OR "teacher" OR "instructor" OR "professor" OR "educator" OR "tutor" OR "lecturer" OR "preceptor") OR ("Faculty"[Mesh])

Individual strategy: Student perception

(("perception"[Mesh]) OR "perception" OR "idea" OR "knowledge" OR "understanding" OR "impression" OR "feeling" OR "opinion" OR "thought" OR "conceptualization") AND (("student"[Mesh]) OR "student" OR "learner" OR "postgraduate" OR "scholar" OR "novice" OR "observer")

Individual strategy: Tools and methods

(Techniques, Educational AND "Teaching"[Mesh]) OR ("education methods" OR "education tools" OR "education procedure" OR "education approach" OR "education technique" OR "education process" OR "education strategy" OR "instructional methods" OR "instructional tools" OR "instructional procedure" OR "instruction approach" OR "instruction technique" OR "instruction process" OR "instruction strategy" OR "education model" OR "instructional model" OR "training model" OR "teaching materials" OR "educational materials" OR "instructional materials" OR "educational techniques" OR "teaching approach" OR "teaching tools" OR "teaching model")

Individual strategy: Emergency transitions

((manage OR management) AND (Disasters[Mesh] OR disaster OR disasters OR emergency OR emergencies))

Final search by student pharmacists without librarian intervention.

((("Emergencies"[Mesh]) OR ("Disasters"[Mesh])) OR ("disaster" OR "disasters" OR "emergency" OR "emergencies" OR "catastrophe" OR "calamity" OR "tragedy" OR "tribulation" OR "fiasco" OR "emergency planning" OR "emergency preparedness" OR "disaster planning" OR "emergency response" OR "crisis management")) AND (Internship [Mesh])) OR ("rotation" OR "rotations" OR ("advanced practice" OR "advanced practice" OR "experiential learning" OR "experiential practice" or "internship" OR "clinical experience" OR "clinical internship" OR "clinicals")) AND ("Pharmacy"[Mesh])) OR (("pharmacy"))) AND (("Education, Distance"[Mesh]) OR ("distance education" OR "remote learning" OR "remote teaching" OR "distance learning" OR "distance teaching" OR "remote education" OR "satellite campus"))
