# Supplementary material for: Implementation of the cognitive apprenticeship model for enhancement of advanced searching skills in a pharmacy academia rotation
Source: J Med Libr Assoc. 2022 Jan 1;110(1):119–25. doi: 10.5195/jmla.2022.1108 (PMC8830337; doi:10.5195/jmla.2022.1108)
Supplement: Supplementary file 4 — Appendix 4. Self-assessment results [file jmla-110-1-119-s04.docx]

**Appendix 4. Self-assessment results.**

|  | Student A | Student B | Student C | Student D |
| --- | --- | --- | --- | --- |
| Searching systematically | Pre Developing | Pre Absolute beginner | Pre Novice | Pre Absolute beginner |
|  | Post Competent | Post Developing | Post Developing | Post Developing |
| Using controlled vocabulary | Pre Developing | Pre Absolute beginner | Pre Absolute beginner | Pre Novice |
|  | Post Competent | Post Developing | Post Competent | Post Developing |
| Creating a reproducible, multi-concept search strategy | Pre Developing | Pre Absolute beginner | Pre Absolute beginner | Pre Absolute beginner |
|  | Post Competent | Post Developing | Post Competent | Post Developing |
| Translating a search for multiple databases | Pre Developing | Pre Absolute beginner | Pre Absolute beginner | Pre Absolute beginner |
|  | Post Developing | Post Developing | Post Competent | Post Developing |
| Building a library of results in a citation manager | Pre Competent | Pre Absolute beginner | Pre Absolute beginner | Pre Absolute beginner |
|  | Post Competent | Post Developing | Post Competent | Post Developing |
