# Supplementary material for: Implementation of the cognitive apprenticeship model for enhancement of advanced searching skills in a pharmacy academia rotation
Source: J Med Libr Assoc. 2022 Jan 1;110(1):119–25. doi: 10.5195/jmla.2022.1108 (PMC8830337; doi:10.5195/jmla.2022.1108)
Supplement: Supplementary file 3 — Appendix 3. Questions from blinded peer review of search strategies [file jmla-110-1-119-s03.docx]

**Appendix 3. Questions from blinded peer review of search strategies.**

|  | Very Accurate or Yes | Mostly Accurate | Inaccurate or No | Incomplete or N/A |
| --- | --- | --- | --- | --- |
| Research topic is clearly represented in the concept clusters. |  |  |  |  |
| Boolean operators (AND/OR) are used appropriately. |  |  |  |  |
| Concepts are properly nested within parentheses. |  |  |  |  |
| Both MeSH terms and keywords are used. |  |  |  |  |
